# Cyclic loading changes the taproot's tensile properties and reinforces the soil via the shrub's taproot in semi-arid areas, China

**DOI:** 10.1038/s41598-024-52740-x

**Published:** 2024-01-27

**Authors:** Jinghua Hu, Xin Zhang, Maolin Yan, Luyi Bai, Shusen Wang, Bo Wang, Jing Liu, Yong Gao

**Affiliations:** 1 College of Desert Control Science and Engineering, Inner Mongolia Agricultural University, Hohhot, 010019 China; 2 Ministry of Water Resources, Institute of Water Resource for Pasturing Area, Hohhot, 010010 China; 3 Landscape Environment Department, Inner Mongolia Academy of Forestry Science, Hohhot, 010013 China; 4 Soil Ecology Department, Inner Mongolia Research Academy of Eco-Environmental Sciences, Hohhot, 010011 China; 5 College of Geographical Science, Inner Mongolia Normal University, Hohhot, 010028 China

**Keywords:** Restoration ecology, Plant stress responses

## Abstract

This study aimed to reveal the soil reinforcement by shrub root systems after repeated stress from external forces, such as high winds and runoff, for extended periods in the wind-hydraulic compound erosion zone. Using the widely distributed Shandong mine area soil and water-conserving plant species, *Caragana microphylla*, *Hippophae rhamnoides,* and *Artemisia ordosica*, cyclic loading tests were conducted on taproots of the three plant species (1–5 mm diameter) via a TY8000 servo-type machine to investigate the taproots’ tensile properties response to repeated loading–unloading using simulated high wind pulling and runoff scouring. Our study revealed that the tensile force was positively correlated with the root diameter but the tensile strength was negatively correlated under monotonic and cyclic loading of the three plants’ taproots. However, after cyclic loading, the three plant species' taproots significantly enhanced the tensile force and strength more than monotonic loading (*P* < 0.05). The taproot force–displacement hysteresis curves of the three plant species revealed obvious cyclic characteristics. Structural equation modeling analysis revealed that root diameter and damage method directly affected the taproots' survival rate, reflecting their sustainable soil reinforcement capacity. The damage method significantly influenced the soil reinforcement more than the root diameter. Our findings reveal that the plant species' taproots can adapt more to the external environment and enhance their resistance to erosion after natural low perimeter erosion damage, effectively inducing soil reinforcement. Particularly, the taproots of *Caragana microphylla* have superior soil-fixing ability and can be used for ecological restoration.

## Introduction

The climate type in central and western China is monsoonal, with cold and arid winters, hot and rainy summers, and mainly torrential rains. It is a typical wind and water erosion area with a fragile ecological environment. The region is also the wealthiest area in China in terms of coal reserves. The long-term frequent coal mining has formed a large area of hollow spaces in the region, resulting in surface subsidence, ground cracks, land degradation, and other phenomena that directly affect the soil’s moisture and nutrient conditions. Consequently, plants cannot absorb sufficient water and nutrients from the soil, leading to their wilting. This can also damage or cause breakage of the root system, causing plant death and aggravating soil erosion^1^. Recently, by implementing many vegetation restoration measures, wind and gravity erosion development trends have been effectively curbed, which play a role in water conservation and soil reinforcement. Vegetation restoration has become fundamental to improving the area’s ecological environment^2^.

The root system constantly branches, penetrating deep into the ground in close contact with the soil and is essential for direct material exchange and interaction between plants and the soil. Existing studies have revealed that the soil reinforcement capacity depends mainly on the root system’s mechanical properties and root–soil interaction^3,4^. Under wind, hydraulic, and gravity erosion damages, the root systems are subjected to multidirectional loading. As a biological material, the root system’s plasticity causes displacement or deformation along the dynamic direction, mainly influenced by the tensile force^5^. Therefore, the tensile property of the root system is crucial to studying plant consolidation and erosion resistance and enhancing slope stability^6^.

Root tensile properties, depending on the morphology and genetic characteristics of the root system, lead to significant differences. Short and thin roots have higher tensile strength, whereas thick roots are stiffer^7^. Plants with more branches in the root system have better soil reinforcement abilities when resisting external forces^8^. Additionally, chemical components such as cellulose and lignin affect tensile strength^9,10^. On the other hand, the external environment is essential in determining the tensile properties of the root system. Generally, the water content of the root system is negatively correlated with the tensile strength^11^. Thus, there are tensile property differences in the root system of the same plant under different topographical conditions^12^. However, the aforementioned research focused on the response of the root systems of specific plant species to the ultimate load in a particular area. They only simulated transient damage leading to root fractures when extreme weather or natural disasters, such as mudslides and flash floods, occur, which is naturally uncommon. More often, the root system is subjected to the frequent reciprocating action of loads, including repeated swaying by high winds, constant scouring by runoff, and slow subsidence of the ground on the root system for extended periods. These load changes are paroxysmal and continuous. The load is gradually transferred to the roots through the plants' leaves, branches, and stems above the ground. This load is transformed into root deformation and continuously acts on the root-soil interaction so that the surface wind speed and runoff flow rate decrease, and soil loss is reduced. Plants continuously reinforce the soil^13,14^. In this process, the root system is repeatedly subjected to loading–unloading and is fatigued. Existing models are all based on taproots in tension and do not take into account root destruction by cyclic external forces. As a result, the calculation results of the models are high and difficult to apply. The construction of the mechanical root consolidation model should be based on the cyclic loading characteristics of the root system and the root consolidation capacity should be accurately assessed.

Currently, most studies on fatigue performance after cyclic loading at home and abroad are on engineering materials^15,16^. There are only a few reports on the fatigue performance of root systems. Mu et al.^17^ used a universal testing machine to study the fatigue performance of the root systems of common tree species: *Pinus tabulaeformis* and *Platycladus orientalis* in northern mountainous areas. This study revealed that cyclic loading affected the tensile properties of plant root systems; however, there was no change in the interspecies ranking of root tensile strength after cyclic loading. Our group also reported the tensile shear response (the root was subjected to a shear force at the stress point and tensile force at both ends, and the entire root system was subjected to tensile and shear forces) of the root system of *Caragana korshinskii* to cyclic loading^18^. Their study revealed that the root system's ultimate tensile force and strength were significantly enhanced after cyclic loading. Thus, repeated cyclic tensioning leads to changes in the mechanical properties of the plant root material, directly affecting the soil reinforcement performance of the plant root system. However, existing studies have not explored the deformation characteristics of the root system during the cyclic process or their differences from those under a monotonic load. There are apparent interspecies differences in the mechanical properties of different plants owing to the root system composition. Moreover, the processes and characteristics of the response to axial cyclic loading also differ. Clarifying the strength and deformation characteristics of the root systems of various plants under cyclic loading in commonly used soil and water conservation species is critical for evaluating the soil reinforcement and erosion resistance of plants and for controlling soil erosion^19,20^. It is also an important tool for explaining the ecological succession of plant communities and the ecological benefits of plantation forestry in terms of plant root erosion resistance.

Based on the above problems, *Caragana microphylla*, *Hippophae rhamnoides*, and *Artemisia ordosica*, the typical soil and water conservation tree species in semi-arid areas, were considered for this study. Cyclic loading under regular changes was simulated in the laboratory, and the root system was subjected to cyclic stretching by repeated loading–unloading. The effects of cyclic loading on the material's mechanical properties, such as the tensile strength and deformation characteristics of *Artemisia ordosica*, were discussed. This study aimed to enrich the sustainable soil fixation process of the root system and provide a theoretical basis for ecological restoration.

## Materials and methods

### Study location

**Figure 1:**
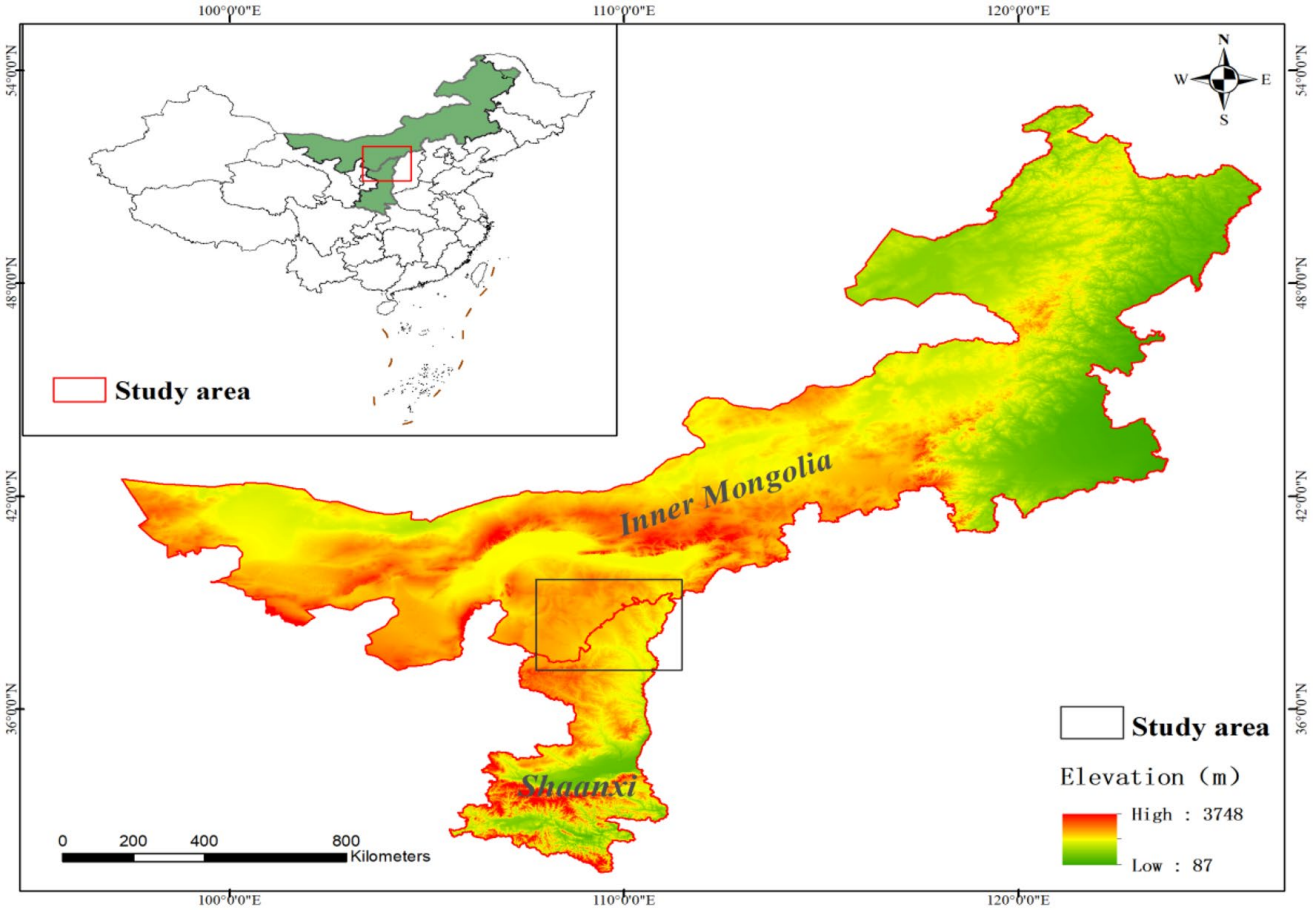
The map of the study area. ^a^The mapping software is ArcGIS 10.7. ^b^URL: https://img01-xusong.91q.com/47CD3C1B-76AB-4EEA-B23D-EA4B9DD09B1E.tif.

The study area was the junction of Yijinhuoluo Banner in Inner Mongolia and Daliuta Town in Shenmu City, Shaanxi Province(Fig. 1), at an altitude of approximately 1000–1300 m and geographical coordinates of E110° 06′–110° 31′, N39° 16′–39° 35′, located in the transition zone between Mu us Sandy Land and Loess Plateau. It is the core area of the Shenfu Dongsheng Coalfield, and the construction area of the Soil and Water Conservation Science and Technology Demonstration Park within the territory is 50 km^2^. This region has a semi-arid continental monsoon climate in the temperate zone, with annual precipitation of 300–500 mm, concentrated and intense rainfall, yearly wind speed of 2.35 m/s, prevailing northwest wind in winter, southeast wind in summer, and sandstorms in winter and spring. The soil type is sandy, has a loose structure, is highly breathable, and has poor water and fertilizer retention ability. The main plant species in the study area include *Caragana microphylla*, *Hippophae rhamnoides*, *Artemisia ordosica*, *Salix psammophila,* and *Amorpha fruticosa*. *Caragana microphylla* has various deep roots, making it suitable for wind and sand control. *Hippophae rhamnoides* are highly adaptable, grow rapidly, and many sprouting branches can quickly cover the sand. *Artemisia ordosica* is a deep-rooted axial-root-type plant with a thick and long root system, which is a good sand-fixing plant. All three species are resistant to wind and sand, more drought-tolerant, and advantageous for ecological management in the study area.

### Root sampling

Communities of *C. microphylla*, *H. rhamnoides*, *and A. ordosica* with good growth and uniformity were chosen in the study area. One hundred plants of each species were randomly selected and measured for height, crown width, and ground diameter, and their mean values were the standard plant index, see Table 1 for details. The root of test plants with parameters close to those of the standard plants were collected. Some roots of the test plants were dug out using the partial excavation method and brought back to the laboratory in cryogenic containers to prevent plant death. The tests were completed within a week of sampling to ensure the test root activity.

**Table 1:** Growth state of standard strains of experimental plants.

Plant species	Density (tree/m^2^)	Coverage (%)	Plant height (cm)	Crown width (cm)		Ground diameter (mm)
				E–W	N–S	
*C. microphylla*	0.13	0.11	136.68 ± 16.59	124.22 ± 23.65	127.79 ± 25.43	12.77 ± 2.53
*H. rhamnoides*	0.31	0.21	125.33 ± 26.25	143.58 ± 30.08	136.54 ± 26.66	27.96 ± 4.32
*A. ordosica*	0.24	0.14	62.27 ± 13.27	73.14 ± 15.92	70.63 ± 14.66	17.50 ± 4.11

**Figure 2:**
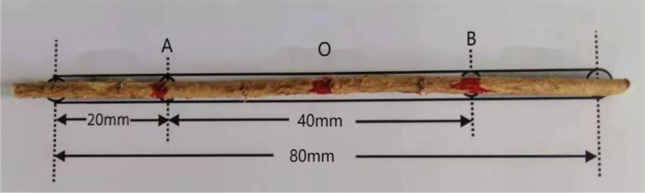
Schematic diagram of test root.

Taproots of *C. microphylla*, *H. rhamnoides*, and *A. ordosica* with diameters < 5 mm accounted for (97.48, 97.89, and 96.92)% of the total root system, respectively. As taproots with root diameter < 1 mm play a biological absorption role rather than a mechanical anchoring role, they contribute less to soil consolidation and improvement. Therefore, 1–5 mm diameter taproots were prepared. The total length of the taproot segment was 8 cm, with 2 cm at each end for the instrument clamping(Fig. 2). The diameters of the test roots were measured at points A, O, and B using an electronic Vernier caliper with an accuracy of 0.01 mm. The diameter of each point was measured twice using the crossover method, and the average value of the three points was the average test root diameter. The relationship between root diameter and tensile strength has been demonstrated in many experiments. This experiment focuses on the effect of different loads on the taproot’s tensile strength for the three plants. Therefore, the workload was reduced without affecting the accuracy, and the diameters were classified into 1–2 mm, 2.5–3.5 mm, and 4–5 mm. Sixty test roots were prepared for each diameter group (30 test roots each were repeated under monotonic and cyclic loads).

### Test design

**Figure 3:**
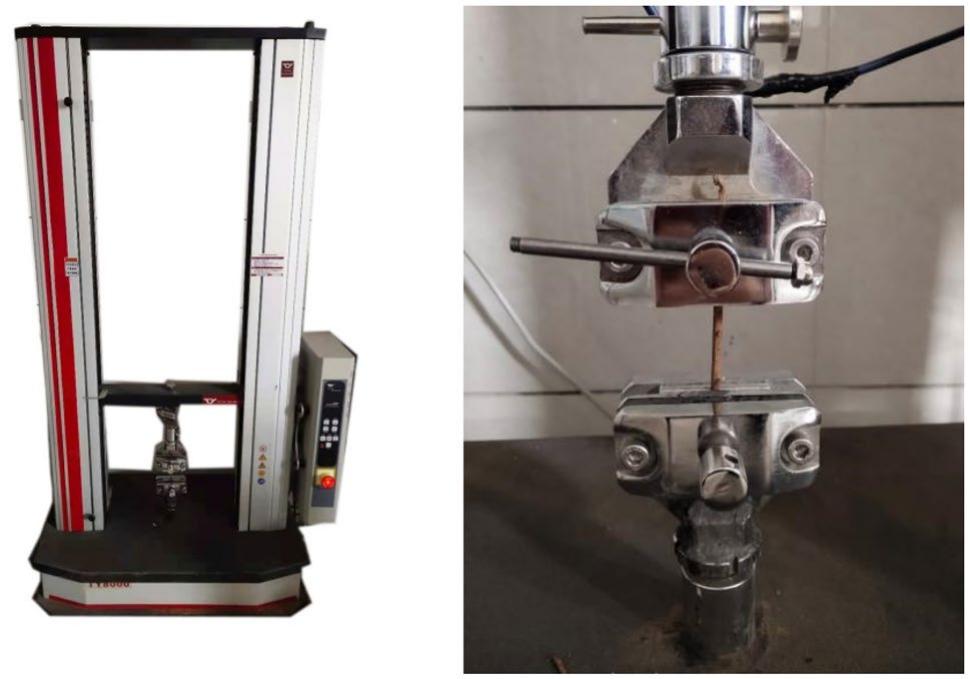
TY-8000 servo control testing machine and fixtures.

This study was conducted in 2021 at the Root Mechanics Laboratory of the College of Desert Management, Inner Mongolia Agricultural University. The 60 test roots prepared for each diameter class group were divided into two groups. Group A was subjected to a monotonic load test, and group B to a cyclic load test(Fig. 3).

Group A: After the test root was subjected to a monotonic load and fracture, the regression equation of its ultimate tensile force and root diameter was fitted.

Group B: Their test root diameters were included in the fitted regression equation of group A, and the fitted ultimate force of each test root of group B was obtained. Subsequently, 70% of the fitted ultimate force was the basis for applying force (the force at which the root systems of the three plants were close to destruction and well above the elastoplastic limit was approximately 70% of the fitted ultimate force). Destructive force was applied to the test roots of group B in cycles, and the root system was pulled off and fitted with the ultimate tensile force-root diameter after completing the cycles. In total, 540 test roots were collected from groups A and B.

### Tensile test

Monotonic and cyclic load tests were performed using a TY8000 servo-control testing machine (Accuracy 0.01 N, speed 1–1000 mm min^−1^). The loading speed was set to 10 mm min^−1^ to observe the root system's force and displacement changes during the cycling process. The prepared test root was placed in the fixture, and the root axis was horizontal. The equipment automatically recorded the tensile force (F) and displacement (S) of the test root during the tensile test. The fracture point was recorded as the wireless data located in the jaw.

#### Monotonic load test

The ultimate tensile force-root diameter regression equation was determined by pulling the test root at a uniform speed until fracture.

#### Cyclic load test

The test root was loaded from 0 N to 70% of the fitted limit force of this test root and then unloaded to 0 N to complete the first cycle, which was repeated. In the pre-test, the stress–strain curve of the root system tended to stabilise after 50 cycles and plastic deformation almost ceased, so the number of cycles in the cyclic test was set at 50. For the 51st cycle, the test root was pulled off, and the time required to conduct a cycle test was approximately 10 min and 40 s.

The Tensile strength is the tensile force per unit cross-sectional area at the damage site, and Eq. (1) is:

1 $$P = 4F/{\uppi }D^{2}$$

where *P* is the ultimate tensile strength (MPa), *F* is the ultimate tensile force (N), and *D* is the test root’s diameter (mm).

### Deformation characteristics

Root elongation was estimated based on the displacement produced by the instrument, which automatically recorded each cycle of the test root in the cyclic test. Equation (2) is:

2 $$\gamma = \Delta L/{\text{L}} \times 100{\text{\% }}$$

where:$\gamma$ is the elongation during the cycle (%), *∆L* is the length of the test root stretched per cycle (mm), and L is the initial length of the test root, which is, the test section (L = 4 cm).

### Constitutive characteristics

The stress–strain curves of the test roots were determined from the ultimate force–displacement curves obtained from the test roots under monotonic and cyclic loading, as indicated in Eqs. (3) and (4). The stress–strain curves were plotted, and the principal curves were analyzed. The stress–strain at the elastic limit of the test root on the constitutive curve is the elastic stress and strain, and the stress–strain when the test root was fractured to withstand the maximum force is the ultimate stress and strain. Subsequently, the elastic modulus was calculated. The elastic modulus is the ratio of the stress and strain at the test root's elastic deformation stage, as indicated in Eq. (5).

3 $$\sigma_{r} = 4F_{{\text{r}}} /{\uppi }D^{2}$$

4 $$\varepsilon_{r} = S_{r} /40 \times 100{\text{\% }}$$

5 $$E_{r} = \sigma_{r} /\varepsilon_{r}$$

where *σ*_*r*_ denotes the stress (MPa), *F*_*r*_ denotes the tensile force (N), and *D* is the average diameter of the test root (mm). In addition, *ε*_*r*_ is the strain (%), and *S*_*r*_ is the displacement of the test root under tension (mm). Finally, *E*_*r*_ is the elastic modulus (MPa).

### Data analyses

The strength, deformation, and constitutive characteristics of the three plants were statistically compiled and fitted to the ultimate tensile force-root diameter and ultimate tensile strength-root diameter relationships using Excel 2010 software. SPSS software (version 20.0) was used to explore plant taproot tensile force and strength variability in the plant species via different loading methods using the least significant extreme difference method. The graphs were drawn using Origin software.

Structural equation modeling (SEM) was performed to identify different hypothetical pathways that explain the association of root diameter and damage method (monotonic and cyclic loading) with the taproot tensile properties of the three plant species responses. Based on prior theoretical knowledge, we hypothesized that root diameter and different damage methods affect the mechanical properties of plant taproots, such as tensile strength, elastic modulus, and deformation, affecting their survival rate. The survival rate of taproots is an essential prerequisite for plants to sustain their soil reinforcement. The insignificant paths in the initial model were gradually removed, and we chose a final model that best fits our data. SEM analysis was performed using the Amos version. The data were fitted into the model using the maximum-likelihood estimation method. Several tests determine the adequacy of the model, including the chi-square (χ^2^) test, goodness of fit index (GFI), and root mean squared error of approximation (RMSEA).

Hierarchical analysis was used to quantify the soil-fixing capacity of taproots of the three plants: *C. microphylla*, *H. rhamnoides,* and *A. ordosica*. We established a comprehensive evaluation index system based on a test of the taproot tensile properties. Weights were assigned to the three plants’ taproot tensile properties, deformation characteristics, and root morphology indices to determine the soil reinforcement index when the plants resisted tensile damage. The hierarchical analysis structure model consisted of three structures: objective layer (O), criterion layer (A), and solution layer (B). The 1–9 scale method determined the degree of importance of the evaluation indicators. Each indicator's and combination weights were calculated according to the judgment matrix. Larger evaluation indexes correlated with stronger soil reinforcement capacity of the plant. The calculation formula is:

6 $$P_{I} = \mathop \sum \limits_{j = 1}^{n} X_{ij} W_{j}$$

where $P_{I}$ is the taproot fixation index and $X_{ij}$ is the standardized value of the jth indicator of the ith plant. $W_{j}$ is the weight value of the jth indicator, and* n* is the number of evaluation indicators.

### Collection of plant material

This study complies with relevant institutional, national, and international guidelines and legislation. The formal identification of *C. microphylla*, *H. rhamnoides*, and *A. ordosica* were carried out by our team through the “Flora of Inner Mongolia”, and a voucher specimen of this material has been deposited in a Chinese Virtual Herbarium(A deposition number of *C. microphylla* is PE 01820622; A deposition number of *H. rhamnoides* is KUN 1447678; A deposition number of *A. ordosica* is PE 02235605). The material of three plants were collected and all methods were performed according to plant materials collection guide of Shaanxi Soil and Water Conservation Science and Technology Demonstration Park.

## Results

### Tensile properties of taproot under the two loads

**Figure 4:**
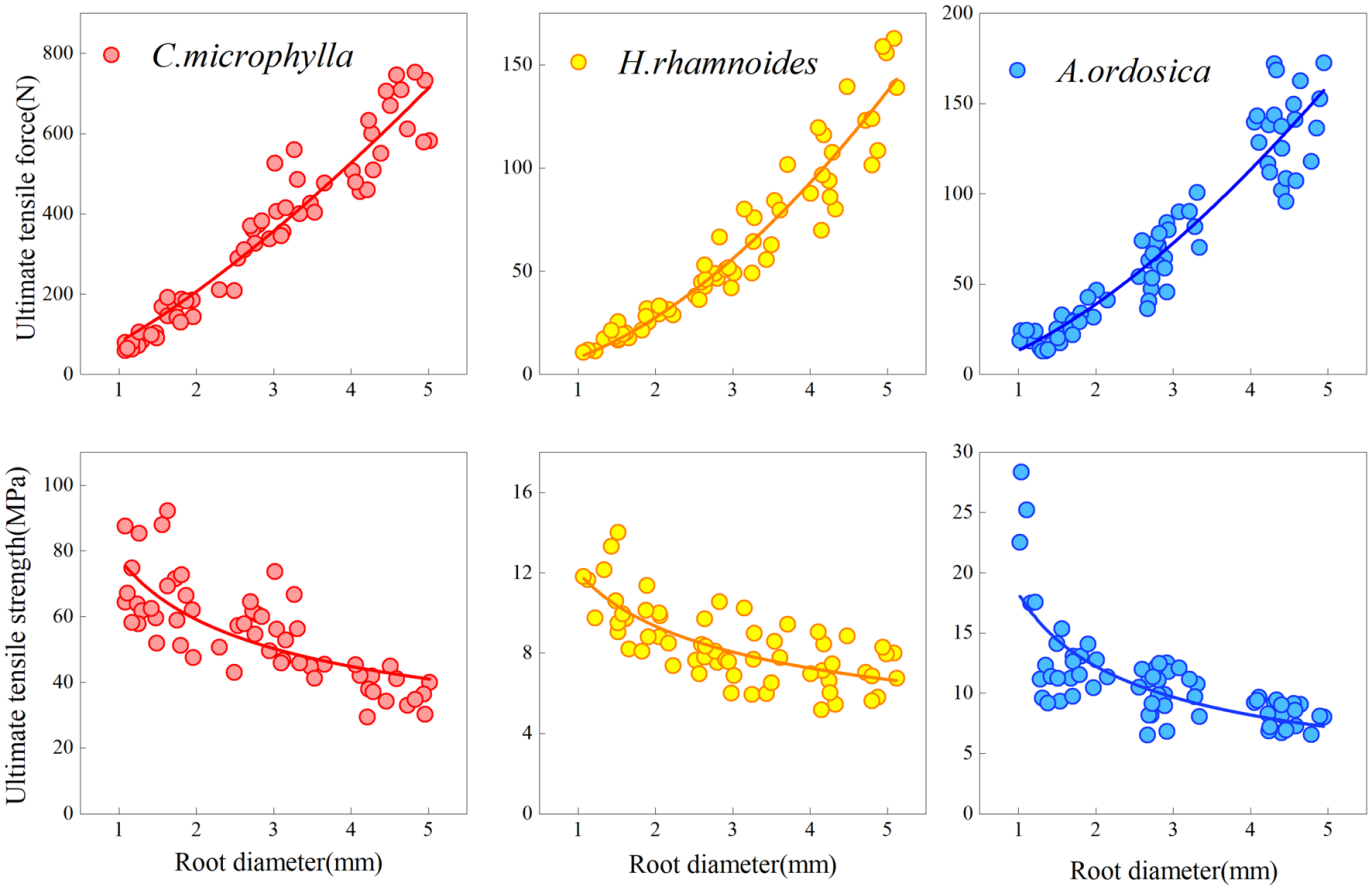
Tensile force-root diameter and tensile strength-root diameter under monotonic loading.

**Figure 5:**
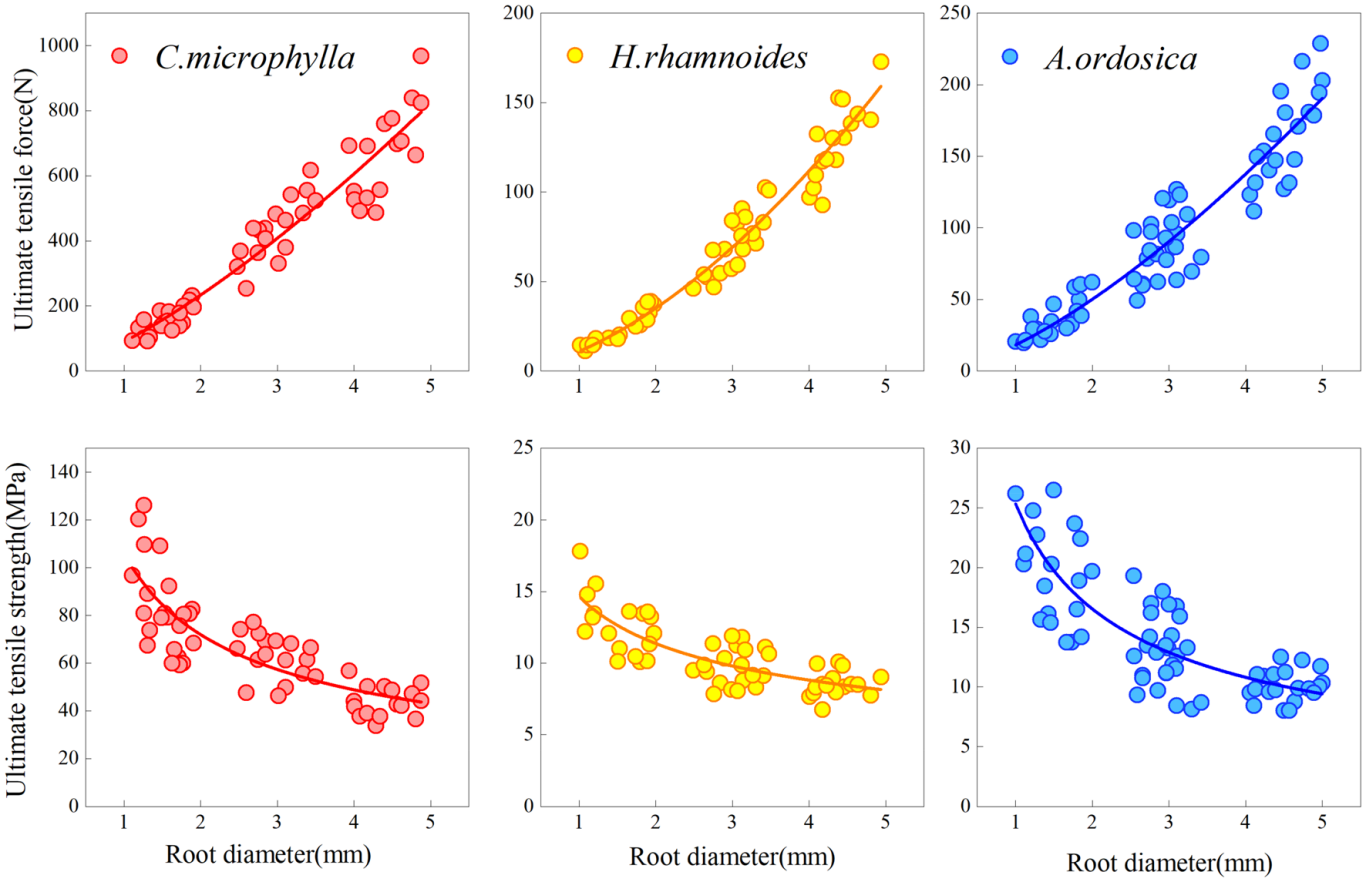
Tensile force-root diameter and tensile strength-root diameter under cyclic loading.

The ultimate tensile force increased with increasing root diameter. The ultimate tensile strength decreased with increasing root diameter for the three species under a monotonic load (Fig. 4). The tensile force and strength correlated as a power function with the root diameter (Table 2). Under 1–5 mm root diameter, *C. microphylla* taproot's range of tensile force and strength were 59.00–752.42 N and 29.45–92.17 MPa, respectively. The tensile force and strength ranges of *H. rhamnoides* taproot were 10.55–162.77 N and 5.16–14.02 MPa, respectively. The tensile force and strength ranges of *A. ordosica* taproot were 12.91–172.60N and 6.55–28.38 MPa, respectively. After cyclic loading, the variation ranges in ultimate root tensile force of *C. microphylla*, *H. rhamnoides*, and *A. ordosica* were 91.17–967.45 N, 11.07–172.69 N, and 19.44–216.69 N, respectively. The variation ranges in tensile strength were 33.72–126.12 MPa, 6.74–17.80 MPa, and 8.00–26.50 MPa. The tensile force-root diameter and tensile strength-root diameter relationships for the three plants were the same as those under monotonic loading (Fig. 5). Besides, cyclic loading did not change their fitted relationships, which remained as power-function relationships. The regression relationships are presented in Table 2. The taproots of the three plants were not pulled off after 50 loading cycles, indicating that the root system could withstand external low circumferential cyclic loading to a certain extent.

**Table 2:** Regressive relationship between root diameter and ultimate tensile properties of different plants.

Loading method	Plant species	Tensile force—root diameter		Tensile strength—root diameter	
		Regression relationship	*R*^2^	Regression relationship	*R*^2^
Monotonic load	*C. microphylla*	*Y* = 62.523 *X*^1.5562^	0.9532	*Y* = 79.607 *X*^−0.444^	0.6234
	*H. rhamnoides*	*Y* = 9.2519 *X*^1.6449^	0.9558	*Y* = 11.78 *X*^−0.355^	0.5021
	*A. ordosica*	*Y* = 12.667 *X*^1.5644^	0.9088	*Y* = 16.449 *X*^−0.478^	0.5522
Cyclic load	*C. microphylla*	*Y* = 81.852 *X*^1.4457^	0.9398	*Y* = 104.22 *X*^−0.554^	0.6966
	*H. rhamnoides*	*Y* = 11.318 *X*^1.6432^	0.9720	*Y* = 14.411 *X*^−0.357^	0.6205
	*A. ordosica*	*Y* = 19.185 *X*^1.4034^	0.9108	*Y* = 24.427 *X*^−0.597^	0.6484

*C. Microphylla* taproot tensile force and strength differed significantly (*P* < 0.05) from those of *H. rhamnoides* and *A. ordosica*, and there was no difference in tensile force and strength between *H. rhamnoides* and *A. ordosica*. Under monotonic and cyclic loads, the interspecific rankings of the taproots' ultimate tensile force and strength were: *C. microphylla* > *A. ordosica* > *H. rhamnoides*. This reveals that under the same root diameter of the three plant species, the root system of *C. microphylla* can withstand a greater degree of external load before fracture. This indicates that this plant species can provide more substantial support and protection to the soil when subjected to the gravitational damage of collapse formed by coal mining subsidence or the pullout damage created by high wind.

**Figure 6:**
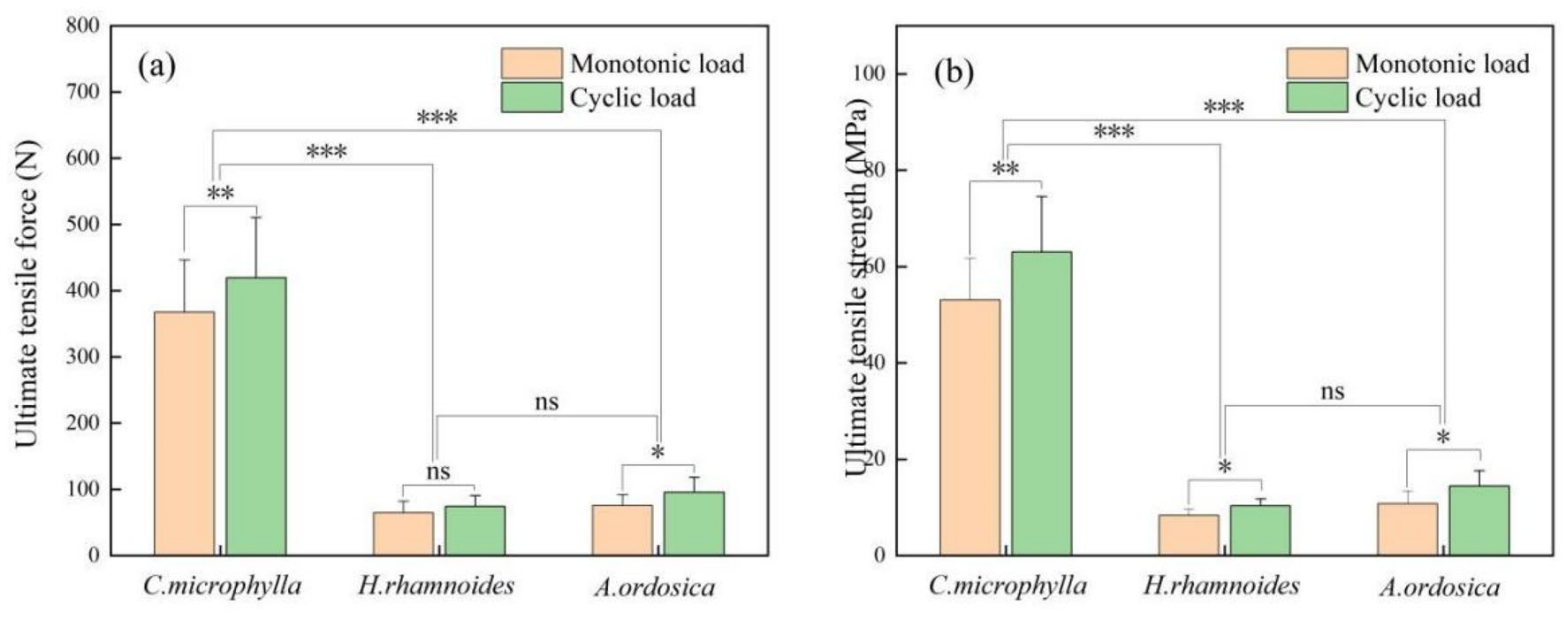
Average tensile force and strength of taproots under monotonic and cyclic loads.

As illustrated in Fig. 4, cyclic loading affected the tensile force (Fig. 6a) and strength (Fig. 6b) of the taproot taproots. The differences in the plant species' taproot tensile force and strength were significant (*P* < 0.05) for both loading methods, except for *H. rhamnoides* taproot tensile force, which did not differ between monotonic and cyclic loading. After 50 loading cycles, the average tensile forces of *C. microphylla* at 1–2 mm, 2.5–3.5 mm, and 4–5 mm root diameters were (150.68, 435.38, and 672.65)N, respectively, which increased by (26.84, 14.74, and 11.22)% compared to monotonic load. The average tensile forces of *H. rhamnoides* were (24.98, 71.33, and 127.92)N, which increased by (12.63, 23.73, and 11.27)%, respectively, compared to the monotonic load. The average tensile strengths of *A. ordosica* were (35.66, 87.48, and 163.81)N, which increased by (41.21, 29.34, and 21.33)%, respectively, compared to the monotonic load. The tensile strength and force displayed a similar regularity; that is, the tensile strength of the taproot after cyclic loading was greater than that under a monotonic load. At three root diameters, the tensile strength increased by (23.16, 15.81, and 14.96)% for *C. microphylla*; (25.35, 23.58, and 20.44)% for *H. rhamnoides*; and (44.88, 27.05, and 23.41)% for *A. ordosica*, respectively, compared to monotonic loading. Overall, the increase in fine root force and strength was incredible. This indicates that plant roots subjected to external low circumferential cyclic loading have enhanced resistance to erosion camping force, which is more conducive to the root system to perform soil reinforcement and erosion resistance.

### Deformation characteristics of the taproot under cyclic loading

Under cyclic loading, the force–displacement curves of the taproots of the three species displayed similar variation patterns. Thus, *A. ordosica* taproots of 2.5–3.5 mm diameter were representative roots (the representative root is the taproot closest to the mean root diameter for that diameter class), and its force–displacement curves were decomposed and analyzed.

**Figure 7:**
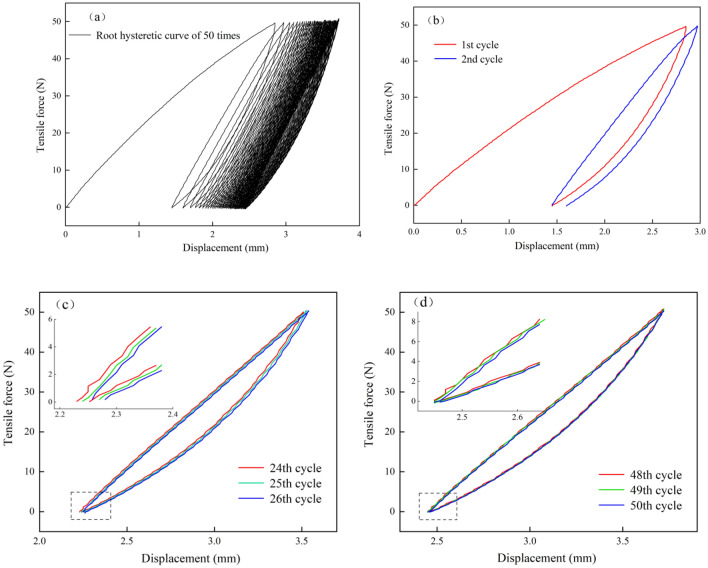
The evolution of the taproot force–displacement curve when subjected to cyclic loading.

The taproot force–displacement curve reveals the apparent periodicity. The curve arrangement changes from “sparse” to “dense” (Fig. 7a). During the cyclic loading process, the force–displacement curve of the taproot during loading was upwardly convex. The force increment under unit displacement gradually decreases. The force–displacement curve is concave when the force is unloaded and does not return along the loading curve, and its deformation exhibits evident hysteresis. Therefore, the loading and unloading section curves form a “hysteresis loop”, in which the first loading–unloading curve is particularly prominent (Fig. 7b). As the number of cycles increases, the “hysteresis loop” becomes incomplete from the initial fuller shuttle shape, the spacing of the hysteresis loop decreases, and the area decreases (Fig. 7c). In the final stage (48th–50th cycles), the hysteresis loop space almost closes. The force–displacement curve continuously repeats on the same cyclic trajectory. At this point, the root system undergoes almost only elastic deformation; therefore, the increment in plastic deformation is negligible. Finally, the hysteresis loop becomes stable (Fig. 7d).

**Figure 8:**
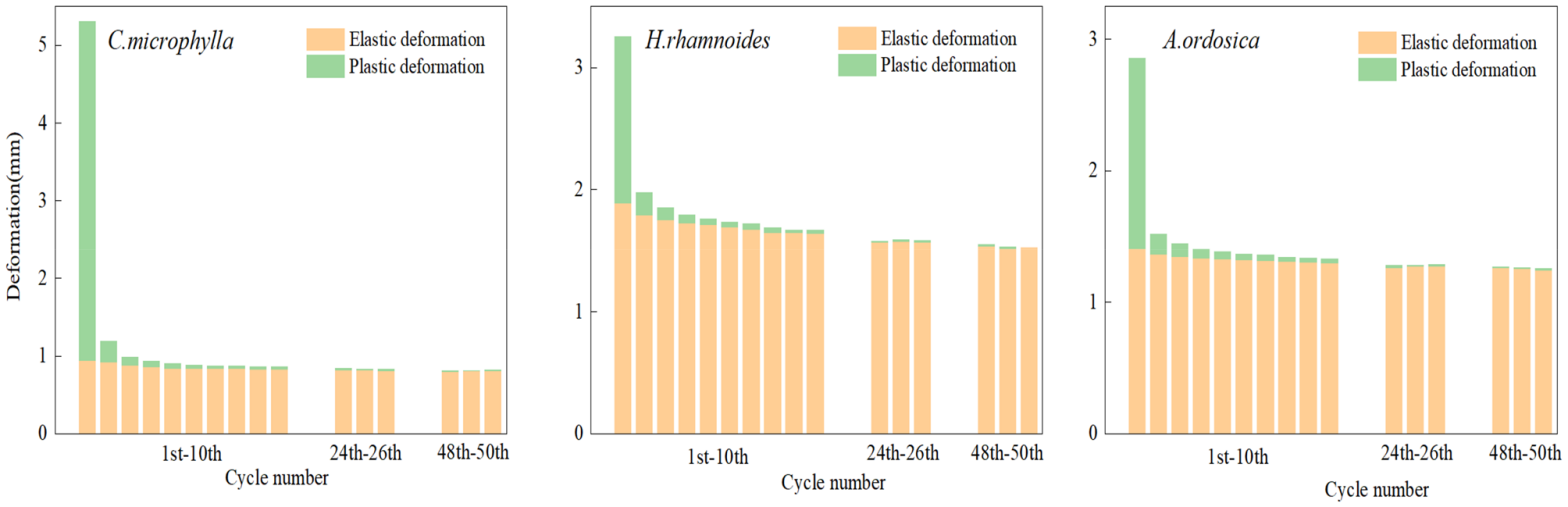
Deformation characteristics of the taproot during the cycle.

Figure 8 quantitatively analyzes the taproot deformation of the three plant species subjected to 50 loading cycles. With each cyclic loading, the taproots of the three plant species produced elastic and plastic deformation, and the taproots had elastic–plastic characteristics. At the first loading, the taproot underwent significant plastic deformation, with (82.10, 41.94, and 50.72)% of the total deformation within the cycle for *C. microphylla*, *H. rhamnoides*, and *A. ordosica*, and (77.33, 58.97, and 58.55)% of the total cumulative plastic deformation, respectively. From the second cycle onward, the plastic deformation of the taproots decreased substantially. The plastic taproot deformation of the three plants in the second cycle accounted for (4.71, 7.78, and 6.19)% of the total accumulated plastic deformation, respectively. The plastic deformation in cycles 1–5 accounted for (86.99, 75.75, and 73.84)% of the total accrued plastic deformation, respectively, the main stage of taproot damage. With an increase in the number of cycles, the total, plastic, and elastic deformation of the taproots of the three plants gradually decreased and stabilized, and the cumulative elongation steadily increased. The elastic percentage fluctuated between 97 and 100%. Generally, each cycle is accompanied by elastic and minimal plastic deformation. Plastic deformation mainly occurs in the early cyclic stress stage. At this stage, the internal structure of the taproot has little strength, and the poor toughness is first destroyed. The magnitude of the change in elastic deformation is not apparent during the entire process, indicating that the strength and toughness of the taproot can continuously reinforce soil through elastic deformation.

### Constitutive characteristics of the taproot limit curve under both loads

**Figure 9:**
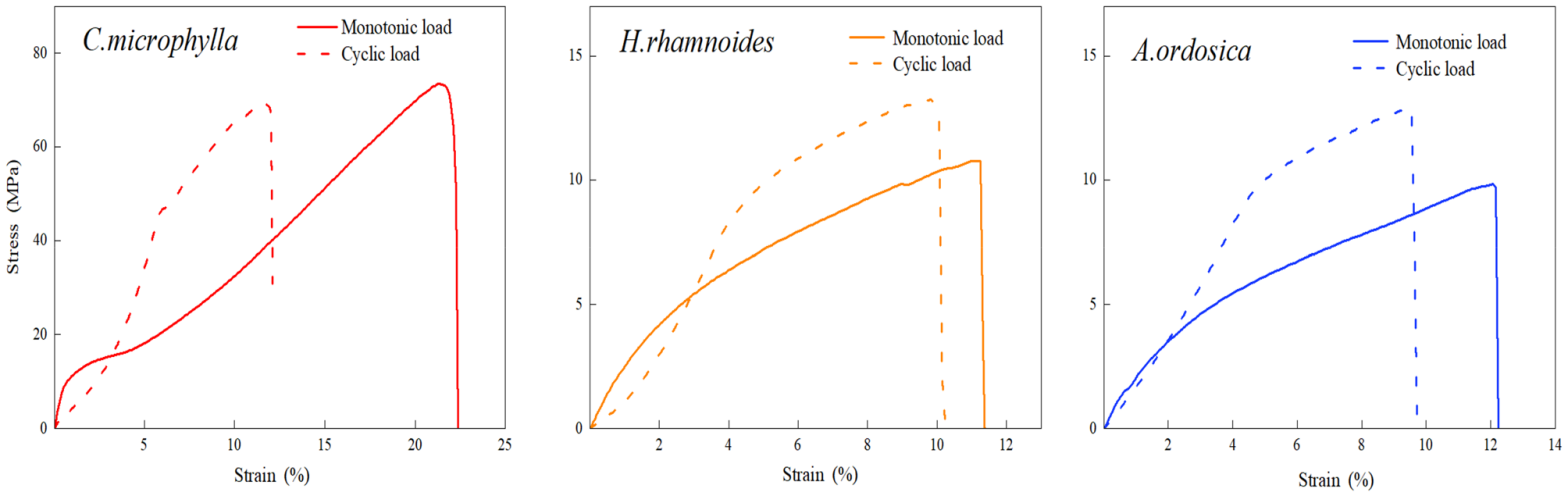
Ultimate stress–strain curves of taproots under both loads.

The ultimate stress–strain curves for the three plant species’ taproots were stressed again until fracture after completing 50 loading cycles, which differed from the curves obtained under a single loading. The differences in the curves of the three plant species’ taproots displayed a consistent regularity under both loading methods. As illustrated in Fig. 9, when subjected to a monotonic load, all parts of the taproot were simultaneously stressed to produce plastic and elastic deformations. The strain increased nonlinearly with increased stress. After the structure with low strength and poor toughness was completely damaged, the taproot was dominated by elastic deformation and the curve leveled. The slope remained constant until the root fractured. The structure with lower strength inside the taproot was damaged during the cyclic process and no longer functioned when resisting external forces. Only elastic deformation occurred at the beginning of the taproot tensile force. In addition, the stress and strain were linearly and positively correlated, similar to the path when loaded during the cyclic process. After reaching the applied load in the cyclic test, the taproot underwent a new plastic deformation. The stress–strain curve slope decreased, similar to the taproot curve under a monotonic load. In general, the ultimate stress–strain curves of the taproots subjected to monotonic and cyclic loads were upwardly convex, meaning that the stress required per unit strain decreased with the gradual destruction of the taproots. However, the elastic properties of the root system were excellent after cyclic loading.

The elastic stress, ultimate stress, and elastic modulus of taproots of the three species were negatively correlated with root diameter. The ultimate strain was positively correlated with root diameter, with no apparent elastic strain regularity (Table 3). Under the same load, the fine roots exhibited better elastic properties and resistance to elastic deformation.

**Table 3:** The taproot’s constitutive characteristic values after monotonic and cyclic loads.

Plant species	Loading method	Root diameter (mm)	Stress (MPa)		Percentage of elastic stress (%)	Strain (%)		Percentage of elastic strain (%)	Elastic modulus (MPa)
			Elastic stress	Ultimate stress		Elastic strain	Ultimate strain		
*C. microphylla*	Monotonic load	1–2	7.64	67.03	11.40	0.53	16.71	3.17	17.39
		2.5–3.5	7.52	54.03	13.92	0.99	18.76	5.28	9.96
		4–5	7.38	38.25	19.29	1.14	23.62	4.83	7.52
	Cyclic load	1–2	18.34	82.55	22.21	3.73	12.28	30.37	7.32
		2.5–3.5	18.22	62.57	29.12	2.92	12.56	23.25	7.05
		4–5	14.69	43.97	33.41	2.89	13.48	21.44	6.91
*H. rhamnoides*	Monotonic load	1–2	3.75	10.10	37.13	1.98	7.44	26.61	5.61
		2.5–3.5	2.57	7.92	32.45	1.16	9.03	12.85	2.78
		4–5	2.46	7.08	34.75	1.30	8.58	15.15	2.42
	Cyclic load	1–2	7.53	12.67	59.43	4.54	9.42	47.34	2.62
		2.5–3.5	5.45	9.79	55.67	4.16	9.50	43.79	1.67
		4–5	5.39	8.53	63.19	4.44	9.53	46.84	1.53
*A. ordosica*	Monotonic load	1–2	4.84	13.95	34.70	1.91	10.67	17.90	3.36
		2.5–3.5	3.77	10.26	36.74	2.50	13.19	18.95	2.27
		4–5	2.89	8.19	35.29	1.63	16.68	9.77	2.22
	Cyclic load	1–2	11.18	20.20	55.35	3.64	8.50	42.82	4.84
		2.5–3.5	7.21	13.03	55.33	3.99	10.34	38.59	2.26
		4–5	5.78	10.11	57.17	3.91	12.04	32.48	1.74

The interspecific relationships between the taproot's elastic stress, ultimate stress, and ultimate strain are: *C. microphylla* > *A. ordosica* > *H. rhamnoides*. The interspecies relationship of elastic strain changed depending on the loading method. However, the elastic strain of *C. microphylla* taproot remained the smallest of the three species. The taproot of *C. microphylla* exhibited deformation characteristics, including high elastic stress, low elastic strain, and high elastic modulus. Under monotonic load, the elastic moduli of *C. microphylla* taproots with 1–2, 2.5–3.5, and 4–5 mm root diameters were (3.10, 3.58, and 3.11), (5.18, 4.39, and 3.39) times higher than those of *H. rhamnoides* and *A. ordosica*, respectively. Under cyclic loading, the elastic moduli of *C. microphylla* taproot were (2.79, 4.22, and 4.51), (1.51, 3.12, and 3.97) times higher than those of the other two, respectively (Table 3). Thus, the taproot of *C. microphylla* can withstand greater forces and undergo more significant deformation by resisting erosion.

The elastic stress, ultimate stress, and elastic strain of the taproots of the three plant species after cyclic loading were greater than those under monotonic loading. The ultimate strain differed in regularity depending on the plant species. At root diameters of 1–5 mm, the mean elastic stresses of *C. microphylla*, *H. rhamnoides*, and *A. ordosica* after cyclic loading were 2.27, 2.09, and 2.10 times higher than those of monotonic loading. The average ultimate stress was 1.19, 1.23, and 1.34 times the monotonic load. In addition, the average elastic strain was 3.59, 2.96, and 1.91 times the monotonic load. The ratio of elastic stress to elastic strain increased accordingly. Due to the variability of elastic potential energy and plastic deformation in different tissue structures of taproots of each plant species, the changes in the cyclic process also vary. There is no apparent regularity of the elastic modulus under various loads (Table 3).

### Evaluation of the taproot tensile property differences of the three plant species

However, the damage method's overall effect on the taproot material's mechanical properties is small and insignificant.The taproot's four material mechanical property variables were related to taproot survival (a prerequisite for sustained soil reinforcement). Root diameter and damage methods also directly and positively affected taproot survival, with damage methods having a significantly more significant impact than root diameter. The final model explained 74% of the total variance in taproots' ability to exert soil reinforcement sustainably.

**Figure 10:**
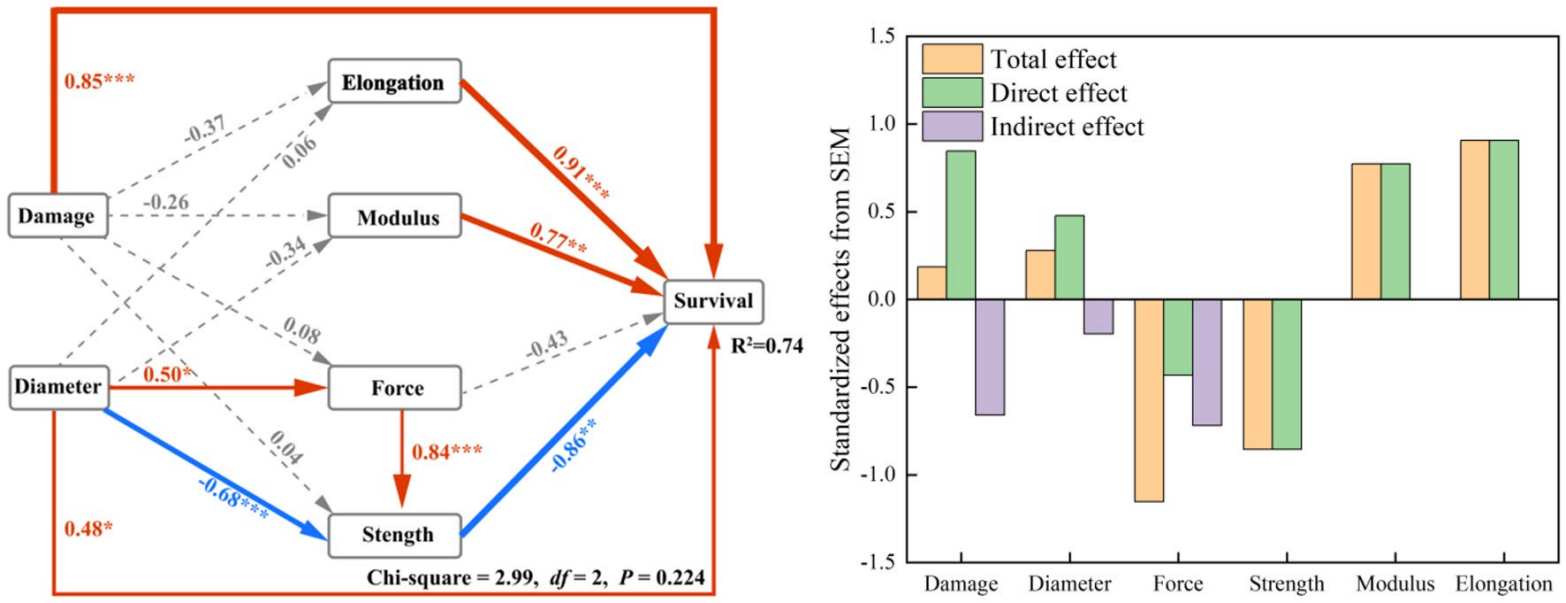
The fitted SEM for the direct or indirect effect of root diameter and damage methods on the taproots' survival. ^a^The model fit the data well: χ^2^ = 2.99, df = 2, *P* = .224, GFI = 0.956, RMSEA = 0.171. Numbers on arrows are standardized path coefficients (equivalent to correlation coefficients). ^b^Asterisks following the numbers represent significant relationships (***P < 0.001,**P < 0.01,*P < 0.05). ^c^Red solid arrows indicate significant positive relationships, solid blue arrows indicate significant negative relationships, and gray dash arrows indicate nonsignificant path coefficients. ^d^Taproot survival rate data from other studies by our group^21^. ^e^Abbreviations: structural equation modeling (SEM), goodness of fit index (GFI), root mean squared error of approximation (RMSEA).

The SEM model was used to identify the primary drivers governing plant taproots to provide continuous soil retention (Fig. 10). SEM analysis indicated that root diameter was the dominant factor influencing the taproot’s strength. The damage methods were the dominant factors influencing the taproot’s deformation characteristics. However, the overall effect of the damage method on the mechanical properties of the taproot material was insignificant, and the mechanical properties of the taproot were related to taproot survival (a prerequisite for sustained soil reinforcement). Root diameter and damage methods also directly and positively affected taproot survival, with damage methods having a significantly greater impact than root diameter. The final model explained 74% of the total variance in the taproots' ability to sustain soil reinforcement.

Three plants’ taproots' tensile performance indices were evaluated, and the main selected indices were tensile properties, deformation properties, and root morphology. All judgment matrices had an overall satisfactory agreement (CR < 0.1).

As illustrated in Table 4, under Instantaneous damage conditions, the soil fixation indices of the three plants were: *C. microphylla* (0.8683) > *H. rhamnoides* (− 0.3831) > *A. ordosica* (− 0.4852). Under cyclic damage conditions, the soil fixation indices of the three plants were: *C. microphylla* (1.0144) > *A. ordosica* (− 0.2591) > *H. rhamnoides* (− 0.7553). The soil reinforcement capacity of *C. microphylla* was the strongest among the three plant species, and it exhibited excellent tensile performance when subjected to transient and cyclic erosion camp forces. It can be the main plant species for ecological restoration in erosional areas dominated by wind and water forces.

**Table 4:** Soil reinforcement index by the three plant species’ taproots under different damage methods.

Damage methods	Soil reinforcement index		
	*C. microphylla*	*H. rhamnoides*	*A. ordosica*
Instantaneous damage	0.8683	− 0.3831	− 0.4852
cyclic damage	1.0144	− 0.7553	− 0.2591

## Discussion

### Strength-diameter relationships

Root diameter, an external representation of the different mechanical properties of taproots, is an essential indicator of the soil-fixing performance of plants. In this study, the ultimate tensile force of 1–5 mm root diameter taproots of *C. microphylla*, *H. rhamnoides*, *A. ordosica* was positively associated with the root diameter as a power function. The ultimate tensile strength was negatively associated with the root diameter as a power function after subjection to 50 loading cycles, similar to the pattern of tensile properties of the root system under a monotonic load. Boldrin^22^, observed the same. The root tensile strength of *Euonymus europaeus* and *Ulex europaeus* plants displayed a negative power function relationship with the root system. The relationships between ultimate tensile force, tensile strength, and root diameter were the same as those under monotonic loading after Lv et al.^23^ had repeatedly loaded and unloaded axial tensile forces 100 and 1000 times, respectively, on the root systems of *Pinus tabulaeformis*. This indicates that different loading methods will not change the root tensile strength-root diameter relationships or affect the fitted relationships. However, some studies have revealed that the relationship between tensile strength and root diameter does not necessarily follow a negative power correlation. However, it is consistent in that relatively finer root systems have better resistance per unit area^24,25^. This phenomenon is mainly due to differences in the chemical content within the root system and their synergistic effect^26^. The chemical composition of the root system includes cellulose, hemicellulose, and lignin. Compared to other chemical components, the cellulose in the root cell wall (responsible for the plant roots’ mechanical strength) is more significant for the degree of mechanical tissue development. It plays a crucial role in plant resistance to external forces^27^. In general, the cellulose content per dry mass of fine root dry matter is greater than that of coarse roots^28^. Hales et al.^29^ also confirmed that cellulose is high in fine roots, and during the gradual thickening of the root diameter, the secondary xylem accumulates outward continuously, gradually compressing the secondary bast, leading to a decrease in root cellulose content. At the same time, microfibrils composed of cellulose molecular chains best resist tension damage, and cellulose increase can enhance the tensile strength of the root system. Therefore, the tensile strength of fine roots is better than that of coarse heels^30,31^. In addition, there was a correlation between lignin content and root diameter. Ye^27^ revealed that the root diameter of *Paspalum natatu* negatively correlates with the lignin content. The reduction in the lignin and hemicellulose content—microfibrils substrates—allows microfibril aggregation, increasing the contact area and improving the root system’s resistance^32^. In summary, chemical composition affects the relationship between plant tensile strength and root diameter. This is independent of the cyclic loading action. Thus, cyclic loading does not change the relationship between the two.

It should be emphasised that although fine roots have superior strength properties, the mechanical properties of very fine roots (root diameter < 1 mm) are negligible. One reason is that, from a function of root tensile force and root system of the three plants, the maximum limiting force of the root system of *C. microphylla* is 46 N when the root diameter is less than 1mm; that of *H. rhamnoides* is 7 N; that of *A. ordosica* is 10 N, all of which are very different from the average tensile force of the root system of each plant. The second reason is the high number of very fine roots, but the average root length is short, making it difficult to form a mechanical complex with the soil, which plays a more absorptive role^33^.

### Effect of cyclic loading on the taproot’s ultimate force and strength

The reciprocal action of erosive forces, such as wind erosion and surface subsidence, frequently acts on plants growing in areas of coal mining collapse. These loads are transmitted to the plant roots, causing fatigue in the root system^34^. As the root system was repeatedly subjected to tensile force in the fatigue state, the internal microstructure of the root system was bound to change. Consequently, the mechanical properties of the root also changed. Our results reveal that the ultimate tensile force and strength of the three plant roots subjected to 50 loading cycles were significantly greater than those under a monotonic load. The variability in the ultimate tensile strength of taproots of *Pinus tabulaeformis* and *Platycladus orientalis* subjected to cyclic tensile loading and after a monotonic tensile load was similar to that of this study by MU et al.^17^. Gai^35^ studied the fatigue performance of taproots of four tree species, including *Larix principis-rupprechtii* and *Quercus mongolica*, and concluded that the ultimate tensile strength of the taproots increased significantly after axial cyclic loading. This indicates that the root system has improved its resistance to external forces and can adapt more to the external environment after repeated subjection to a certain degree of erosion. This may be because, during cyclic loading, the repeated tension of the root system led to the continuous squeezing of the cells for a water content deficit, which enhanced the bonding strength between the organic polymers in the cell walls to the extent that the roots exhibited better tensile properties after cyclic loading^36^. Many studies have reported that high root water content negatively affects root tensile properties. Moreso, Zhu et al^37^ observed that when the root water content increased by approximately 11%, the tensile strength of the root system persistently decreased by approximately 15%. Zhang^38^ also revealed that mild water loss could significantly increase the tensile strength of taproots. On the other hand, cyclic tension and loss of root water shrink the root diameter, and the decrease in root diameter also increases tensile strength^39^. Jaffe's^40^ study revealed that plants are not susceptible to re-injury after mechanical disturbance. In other words, after cyclic loading, the ultimate tensile performance of the root system does not weaken, and it enhances the resistance to erosion forces, which can exert better-sustained soil reinforcement effectiveness. This can also explain the natural succession of pioneer tree species in windswept areas from the point of view of root stress. Different plant root systems have different tolerances to erosion loading, and prolonged, high-frequency erosion can cause root damage in some plant species, affecting survival rates.

### Deformation characteristics

Like most plant roots, the taproots of these three plants are elastoplastic. When subjected to an external load, the root system deforms and rubs against the inter-root soil, converting the external force into the root-soil interface frictional resistance, and reducing the direct effect of external force on the soil, thus stabilizing the soil^41^. During the cyclic test, a hysteresis loop was formed by the taproot loading–unloading curve of the three plants, in which the first loading curve significantly differed from the other cyclic loading curves. From the energy point of view, all the work done by the external force on the taproot during loading is part of the elastic potential energy that causes the root system’s elastic deformation. The other amount of energy causes the local taproot’s plastic deformation so that this part of the energy will not be released from the root system with the unloading of the external force. Thus, the unloading curve lags behind the loading curve, forming a hysteresis loop^23^. During the cyclic process, the taproot force–displacement curve can reflect the deformation process when the taproot is subjected to an external force. The change in the curve is mainly related to the mechanical strength exerted by each component inside the root system to resist tensile damage. The structure of the root system from outside to inside is the periderm, secondary phloem, and secondary xylem. There are differences in the mechanical strengths of each component^42^. At the first loading, the taproot plastic deformation accounted for 58.55% of the accumulated plastic deformation and only 6.19% at the second loading, indicating that when the root system was subjected to tensile action, all parts of the root system were stressed simultaneously. A periderm with lower strength and poor toughness is the first to be damaged at the first loading. It loses resistance to external forces^43^. As the number of cycles increased, the taproot was deformed elastically, with minimal plastic deformation. The periderm has been destroyed; therefore, this stage is played by the secondary phloem and xylem together. The secondary phloem and xylem inside the taproot can slow down root damage by the tensile action of the fiber filaments under the tensile act^44^. Under constant cyclic load, the plastic deformation produced by multiple stresses gradually changed the equilibrium position of the stress distribution of the taproot. The fiber filaments are unevenly stressed and progressively fractured, slowly accumulating damage. The fiber bundles are progressively damaged^45^. Therefore, the taproot of the three plants could withstand a certain degree of external low circumferential load under the premise of soil reinforcement performance.

## Conclusion

The natural cyclic loading effects leading to soil erosion were simulated indoors to study the response of the plant’s taproot tensile properties to cyclic loading. The root systems of three species of plants, *C. microphylla*, *H. rhamnoides*, and *A. ordosica*, were damaged using the low circumferential erosion camp forces of nature, such as wind blowing and hydraulic scouring. Instead of weakening, the ultimate tensile strength of the plant root system was more conducive to enhancing the soil reinforcement capacity. The root diameter and damage method directly and positively influenced the sustained soil fixation capacity of the three species’ taproots. In contrast, the damage method significantly influenced the fixation capacity of taproots more than the root diameter. However, root diameter remained the dominant factor affecting the mechanical properties of the taproots. Therefore *C. microphylla* is the preferred plant species for ecological restoration in areas with wind- and water-dominated erosion.

## Data Availability

The datasets generated during and analysed during the current study are available from the corresponding author on reasonable request.

## Abbreviation

SEM: Structural equation modeling
